# Genomic analysis and characterization of phages infecting the marine *Roseobacter* CHAB-I-5 lineage reveal a globally distributed and abundant phage genus

**DOI:** 10.3389/fmicb.2023.1164101

**Published:** 2023-04-17

**Authors:** Zefeng Zhang, Zuqing Wu, He Liu, Mingyu Yang, Rui Wang, Yanlin Zhao, Feng Chen

**Affiliations:** ^1^Institute of Marine Science and Technology, Shandong University, Qingdao, China; ^2^Fujian Provincial Key Laboratory of Agroecological Processing and Safety Monitoring, College of Life Sciences, Fujian Agriculture and Forestry University, Fuzhou, China; ^3^Institute of Marine and Environmental Technology, University of Maryland Center for Environmental Science, Baltimore, MD, United States

**Keywords:** CHAB-I-5, roseophage, genomics, phylogenetic analysis, metagenomic viral genomes, novel phage genus, biogeography

## Abstract

Marine phages play an important role in marine biogeochemical cycles by regulating the death, physiological metabolism, and evolutionary trajectory of bacteria. The *Roseobacter* group is an abundant and important heterotrophic bacterial group in the ocean, and plays an important role in carbon, nitrogen, sulfur and phosphorus cycling. The CHAB-I-5 lineage is one of the most dominant *Roseobacter* lineages, but remains largely uncultured. Phages infecting CHAB-I-5 bacteria have not yet been investigated due to the lack of culturable CHAB-I-5 strains. In this study, we isolated and sequenced two new phages (CRP-901 and CRP-902) infecting the CHAB-I-5 strain FZCC0083. We applied metagenomic data mining, comparative genomics, phylogenetic analysis, and metagenomic read-mapping to investigate the diversity, evolution, taxonomy, and biogeography of the phage group represented by the two phages. The two phages are highly similar, with an average nucleotide identity of 89.17%, and sharing 77% of their open reading frames. We identified several genes involved in DNA replication and metabolism, virion structure, DNA packing, and host lysis from their genomes. Metagenomic mining identified 24 metagenomic viral genomes closely related to CRP-901 and CRP-902. Genomic comparison and phylogenetic analysis demonstrated that these phages are distinct from other known viruses, representing a novel genus-level phage group (CRP-901-type). The CRP-901-type phages do not contain DNA primase and DNA polymerase genes, but possess a novel bifunctional DNA primase-polymerase gene with both primase and polymerase activities. Read-mapping analysis showed that the CRP-901-type phages are widespread across the world’s oceans and are most abundant in estuarine and polar waters. Their abundance is generally higher than other known roseophages and even higher than most pelagiphages in the polar region. In summary, this study has greatly expanded our understanding of the genetic diversity, evolution, and distribution of roseophages. Our analysis suggests that the CRP-901-type phage is an important and novel marine phage group that plays important roles in the physiology and ecology of roseobacters.

## 1. Introduction

As the most abundant and diverse entities in the world’s oceans, viruses play critical roles in driving marine biogeochemical cycles and shaping the community structure and function of marine microorganisms (Suttle, 2007; Breitbart, 2012). Marine viruses are responsible for 20% to 40% of microbe mortality, therefore, shaping the composition and functional processes of marine microbial communities (Suttle, 2007; Breitbart, 2012). Viral-mediated cell lysis directly affects the marine carbon cycle by releasing ~10 billion tons of carbon from cellular organisms into the marine environment every day (Suttle, 2007; Breitbart, 2012). In addition, viruses drive microbial evolution and diversification by serving as selective pressure and key mediators in horizontal gene transfer.

Marine viral communities have tremendous genetic diversity (Roux et al., 2016; Gregory et al., 2019). In the last decade, culture-independent viromic surveys have generated a vast number of sequencing data and provided unprecedented insight into the genetic diversity and potential metabolic functions of marine viral communities. Furthermore, a large number of new viral populations have been identified (Brum et al., 2015; Roux et al., 2016; Gregory et al., 2019). However, due to the lack of culturable representatives, more than 80% of marine virome sequences are regarded as “dark matter” that has not yet been annotated, which is a major challenge in viromic studies (Brum et al., 2015; Roux et al., 2016; Gregory et al., 2019). The lack of culturable representatives also limits our knowledge of the infection host, their impact on hosts and ecosystems, and the validation of metagenomic results. Currently, less than 1% of marine phages have been isolated and cultured in the laboratory (Brum et al., 2015; Roux et al., 2016). In this regard, more phages need to be isolated to help improve the annotation and characterization of metagenomic data.

The *Roseobacter* group in the *Alphaproteobacteria* is one of the most abundant marine bacterial groups, comprising up to 20% of the bacteria cells in coastal waters and 3% to 5% in open ocean surface waters (Wagner-Dobler and Biebl, 2006; Moran et al., 2007; Brinkhoff et al., 2008). The *Roseobacter* group has great genomic diversity and various metabolic capabilities, thus playing important roles in global biogeochemical cycles (Wagner-Dobler and Biebl, 2006; Brinkhoff et al., 2008; Lidbury et al., 2014; Luo and Moran, 2014). Members of the *Roseobacter* group are extremely diverse, comprising 141 distinct subclusters at the genus level (Buchan et al., 2005; Wagner-Dobler and Biebl, 2006; Brinkhoff et al., 2008; Luo and Moran, 2014; Pujalte et al., 2014). Although many roseobacters could be cultured in the laboratory, most cultured roseobacters are systematically different from those in nature in terms of genome composition, G + C content, and base composition (Luo et al., 2012; Luo and Moran, 2014). Metagenomic data analysis based on 16S rRNA gene showed that the dominant roseobacters in the ocean are mainly present in a few *Roseobacter* lineages, including CHAB-I-5, SAG-O19, DC5-80-3(RCA), NAC11-7, and other lineages (Buchan et al., 2005, 2009; Zhang et al., 2016). Among these dominant *Roseobacter* lineages, the CHAB-I-5 lineage is present in various oceanic areas, representing ~20% of roseobacters and ~6% of bacterioplankton in some areas (Buchan et al., 2005; Billerbeck et al., 2016; Zhang et al., 2016). Despite its ecological dominance, only one CHAB-I-5 strain, SB2, has been isolated and reported thus far (Billerbeck et al., 2016).

Currently, 46 roseophages infecting six different *Roseobacter* subclades have been isolated and reported (Bischoff et al., 2019; Cai et al., 2019; Zhan and Chen, 2019; Zhang et al., 2019; Ma et al., 2021; Rihtman et al., 2021; Zhai et al., 2021). Most roseophages isolated from roseobacters capable of growing rapidly in nutrient-rich media do not represent the dominant *Roseobacter* lineages in the ocean (Bischoff et al., 2019; Cai et al., 2019; Zhan and Chen, 2019; Ma et al., 2021; Rihtman et al., 2021). Due to the difficulty in culturing the hosts, little is known about phages infecting dominant *Roseobacter* lineages. Two recent studies have reported nine phages infecting *Roseobacter* RCA strains, which belong to five distinct phage groups, indicating that various phages could infect RCA bacteria (Zhang et al., 2019; Zhai et al., 2021). Unlike other isolated roseophages, these RCA phages are more widely distributed and have higher relative abundances in the ocean (Zhang et al., 2019). The isolation of RCA phages emphasizes that studying phages infecting dominant roseobacters could greatly expand our knowledge of marine roseophages and promote the annotation and understanding of metagenomic data.

This study used a new CHAB-I-5 bacterium, FZCC0083, as a host to isolate two CHAB-I-5 phages. The genomic analysis revealed the genomic novelty of the two roseophages. We also identified some metagenomic viral genomes (MVGs) closely related to the two CHAB-I-5 phages. Finally, metagenomic analysis revealed their prevalence in the coastal, estuarine, and polar waters.

## 2. Materials and methods

### 2.1. The cultivation, 16S rRNA gene sequencing of CHAB-I-5 strains

The *Roseobacter* strain FZCC0083 was isolated from the coastal waters of Pingtan Island (lat.’ N25°26′, long. E119°47′) on 13 May 2017 using the dilution-to-extinction method (Stingl et al., 2007). The culture medium was prepared using the autoclaved natural seawater supplemented with 1 mM NH_4_Cl, 100 μM KH_2_PO_4_, 1 μM FeCl_3_, a mixed carbon source (Cho and Giovannoni, 2004), and vitamins (Sun et al., 2011). The strain FZCC0083 was incubated at 23°C. The 16S rRNA gene sequence of FZCC0083 was PCR amplified from cultures with the 16S rRNA gene primers 27F and 1492R (Lane, 1991) and sequenced by Sanger sequencing.

### 2.2. Source waters and CHAB-I-5 phage isolation

The seawater samples used to isolate CHAB-I-5 phages were collected from the surface waters of the North Sea (lat.’ N53°56′, long. E7°48′) and the Yellow Sea (lat.’ N36°38′, long. E121°10′; Table 1). Before use, the seawater samples were filtered through a 0.1 μm filter to remove the cellular organisms and stored at 4°C. Phage isolation was performed using a previously reported liquid medium-based isolation method (Zhao et al., 2013). Briefly, the filtered seawater sample was incubated with the FZCC0083 cultures in the exponential growth phase. The growth of FZCC0083 during incubation was monitored using a Guava EasyCyte flow counter (Merck Millipore, Billerica, MA, United States) with SYBR Green I (Invitrogen, Eugene, OR, United States). Cell lysis cultures were collected and filtered using a 0.1 μm filter to remove the cellular organisms. Phages were purified using the dilution-to-extinction method.

**Table 1:** General features of the two CHAB-I-5 phages analyzed in this study.

Phage	Original host	Source water	Depth	Latitude	Longitude	Date of collection	Genome size (bp)	% G + C	No. of ORFs	Accession no
CRP-901	FZCC0083	North Sea	6 m	N53°56′	E7°48′	March 2019	53,013	45.57	77	OQ401623
CRP-902	FZCC0083	Yellow Sea	Surface	N36°38′	E121°10′	September 2021	51,954	45.50	80	OQ401624

### 2.3. Phage DNA preparation, genome sequencing, and genome assembly

250 mL of each phage lysate were filtered through 0.1 μm filters and concentrated using Amicon Ultra centrifugal filters (30 kDa; Merck Millipore). Phage DNA was extracted using the formamide extraction method (Sambrook and Russell, 2001). The genomes of the phages were sequenced using the Illumina paired-end HiSeq 2500 sequencing approach (2 × 150 bp) at Beijing Novogene Technology (Beijing, China). The NEBNext Ultra™ DNA Library Prep Kit for Illumina (New England Biolabs, USA) was used to construct the DNA sequencing library. Each sample generated at least 2 Gb of raw data. The raw sequencing data were quality filtered using FASTP v0.20.1 (-q 20 -l 50 -w 15; Chen et al., 2018), including removing sequencing adapters, low-quality sequences, and short sequences. The filtered reads were assembled *de novo* using megahit v1.2.9 with the default settings (Li et al., 2016). The genome of CRP-902 was assembled into a circular with terminal repeats. A 52.9 kb CRP-901 contig was assembled and the gap was closed by PCR amplification using a pair of primers (F: 5′-ACTGGAATAAGCCCAGTCGC-3′, R: 5′-TGATCCATCGCGTGTGCTAA-3′). The PCR reaction consisted of an initial denaturation at 94°C for 3 min, 35 cycles of denaturation at 94°C for 30 s, annealing at 55°C for 30 s, and elongation at 72°C for 2 min.

### 2.4. Genome annotation and comparative genomic analysis

Genes of the phage genomes were predicted using Prokka v1.14.6 (--metagenome --kingdom Viruses --gcode 11 --evalue 0.001; Seemann, 2014), RAST server (Aziz et al., 2008), and manual inspection. tRNAs were predicted using the tRNAscan-SE server (Lowe and Eddy, 1997). The biological functions of the putative ORFs were annotated using BLASTp (amino acid identity ≥ 25%, alignment coverage ≥ 50%, and e-value ≤ 1E-3) against the NCBI non-redundant (nr) database, NCBI-RefSeq database, and the NCBI virus database for comparison with known proteins.

The protein homologs of ORFs were identified using hmmscan (-E 0.001) in HMMER v3.3 (Eddy, 2011) against the Pfam-A database. The average nucleotide identity (ANI) and average amino acid identity (AAI) between the phage genomes were calculated using fastANI v1.32 (Jain et al., 2018) and EzAAI v1.2.2 (Kim et al., 2021), respectively.

### 2.5. Metagenomic recruitment of marine MVGs closely related to CRP-901 and CRP-902

The marine MVGs were extracted from the IMG/VR database v3 (Roux et al., 2021), Pearl River estuary database (Xu et al., 2022), Red Sea database (Hevroni et al., 2020), sequences assembled from 14 coastal stations (Tsiola et al., 2020), sequences assembled from the 78 marine viromes (Coutinho et al., 2017), MedDCM fosmid library (Mizuno et al., 2013), Station ALOHA assembly free virus genomes (Beaulaurier et al., 2020), and ALOHA 2.0 viromic database (Luo et al., 2020). In order to recruit MVGs closely related to CRP-901 and CRP-902, the protein sequences of the four conserved genes of CRP-901 and CRP-902 were aligned using MUSCLE, including bifunctional DNA primase-polymerase, DNA helicase, capsid, and terminase large subunit (TerL). The Hidden Markov model (HMM) of the four conserved genes was constructed using hmmbuild in HMMER (Eddy, 2011; Supplementary Data 1). The constructed HMM was used to query the marine MVGs using hmmsearch (−E 0.001). The matched sequences were further compared with the four conserved genes by BLASTp (-evalue 1e-3 -qcov_hsp_perc 50). Only matches with ≥50% alignment length, ≥25% identity, and ≥50 bitscore were considered homologous. Furthermore, only MVGs containing at least one of the bifunctional DNA primase-polymerase homolog or DNA helicase homolog and at least one of the capsid homolog or TerL homolog were retained. The completeness and quality of recruited MVGs were assessed using CheckV v0.8.1 (end_to_end; Nayfach et al., 2021) and 395 MVGs with genome completeness > 50% were used for further analysis. vConTact 2.0 (--db “None” --rel-mode ‘BLASTp’ --blast-evalue 0.001--c1-bin cluster_one-1.0.jar --optimize) was used to calculate the similarity score between every pair of the 395 MVGs, CRP-901, CRP-902, and other known viruses from NCBI-RefSeq (v212), and ClusterONE (Nepusz et al., 2012) was used to identify the viral clusters with the default parameters which are defined in the vConTACT 2.0 (Supplementary Table 1). A total of 24 MVGs were clustered into the same viral cluster with CRP-901 and CRP-902, and these 24 MVGs were retrieved for further analysis.

Genes of the 24 MVGs were predicted by Prokka v1.14.6 (--metagenome --kingdom Viruses --gcode 11 --evalue 0.001; Seemann, 2014). The orthologous gene groups of CRP-901, CRP-902, and the 24 MVGs were identified by OrthoFinder (Emms and Kelly, 2015) using all-*vs*-all BLASTp (amino acid identity ≥ 25%, alignment coverage ≥5 0%, and e-value ≤1E-3). The biological functions of the ORFs were annotated with BLASTp (amino acid identity ≥25%, alignment coverage ≥50%, and e-value ≤1E-3) against the NCBI non-redundant (nr) database, NCBI-RefSeq database, and the NCBI virus database for comparison with known proteins. The ANI and AAI between CRP-901, CRP-902, and the 24 MVGs were calculated using fastANI v1.32 (Jain et al., 2018) and EzAAI v1.2.2 (Kim et al., 2021), respectively.

### 2.6. Phylogenetic analysis

The 16S rRNA gene sequences of the known roseobacters and FZCC0083 were aligned using MUSCLE (Edgar, 2004) with the default parameter. The optimal amino acid substitution models of alignments were evaluated using IQ-TREE v1.6.12 (-m MF; Minh et al., 2020). The 16S rRNA gene phylogenetic tree was constructed using IQ-TREE with 1,000 bootstrap replicates.

The genome-wide proteomic tree between CRP-901, CRP-902, other known prokaryotic dsDNA phages from the ViPTree server, and 40 related dsDNA phages (listed in Supplementary Table 2) was constructed using the ViPTree server (Nishimura et al., 2017), which was calculated by tBLASTx for genome-wide sequence similarities. To evaluate the evolutionary relationship between the 24 MVGs, CRP-901, and CRP-902 and to determine their taxonomic positions, we constructed a whole-genome phylogenetic tree based on amino acid sequences with the Virus Classification and Tree Building Online Resource (VICTOR, https://ggdc.dsmz.de/victor.php; Meier-Kolthoff and Goker, 2017) using the Genome-BLAST Distance Phylogeny method with 100 bootstrap replicates and formula *d_0_*. We also evaluated the phages at genus, subfamily, and family levels using the OPTSIL program (Goker et al., 2009). We constructed maximum likelihood phylogenetic trees of bifunctional DNA primase-polymerase, DNA helicase, capsid, and TerL. Sequences were aligned using MUSCLE v3.8.1551 (Edgar, 2004) with the default parameter and trimmed by trimAL v1.4.rev15 (Capella-Gutierrez et al., 2009) using the automatic mode (−automated1). The optimal amino acid substitution models of alignments were evaluated using IQ-TREE v1.6.12 (-m MF; Minh et al., 2020), and the maximum likelihood trees were constructed by IQ-TREE v1.6.12 (-bb 1,000 -alrt 1,000 -nt AUTO) using the optimal substitution model with 1,000 bootstrap replicates.

### 2.7. Host prediction

The potential hosts of the 24 MVGs were predicted using the RaFAH tool v0.3 with default settings (--predict; Coutinho et al., 2021), which predicts host information based on protein content using the random forest method. A trained and validated random forest model was built using CRP-901, CRP-902, and other known viruses downloaded from the NCBI RefSeq database (v212).

### 2.8. Metagenomic read-mapping analyses

A total of 258 marine virome datasets were used for metagenomic read-mapping to assess the relative abundance of phages, including Global Ocean Viromes (v2.0; Gregory et al., 2019), Pearl River estuary virome (Xu et al., 2022), Mariana Trench virome (Gao et al., 2022), Eastern Tropical North Pacific virome (Jurgensen et al., 2022), viromes of the Delaware Bay and Chesapeake Bay (Sun et al., 2021), Black Sea virome (Jaiani et al., 2020), Red Sea virome (Hevroni et al., 2020), South China Sea DNA virome (Liang et al., 2019), Pacific Ocean Virome (Hurwitz and Sullivan, 2013), Scripps Pier Virome (Hurwitz et al., 2013), and India Ocean Virome (Williamson et al., 2012; listed in Supplementary Table 3). Duplicate sequences of the 24 MVGs were removed using CD-HIT v4.8.1 (-c 0.95 -aS 0.8), and the longest sequences of each species cluster were retained for read-mapping analysis. Viromic reads were mapped against the non-redundant phage genomes using coverm v0.6.1 (-p bwa-mem --min-read-percent-identity 95 --min-read-aligned-length 50). The relative abundances of phages were normalized by mapped read counts per kilobase pair of genomes per million read counts in the metagenome (RPKM). Phages were considered present and abundance values were retained if their genome coverage was >40% in the data set (Buchholz et al., 2021; Qin et al., 2022). Differences in the abundance of viruses were compared using the Mann–Whitney *U*-test in R v4.1.2 and plotted with the pheatmap R package. Correlations between phage abundance and environmental factors were evaluated using linear regression in R.

## 3. Results and discussion

### 3.1. Host strain and general features of CHAB-I-5 phages

The 16S rRNA gene sequence of host strain FZCC0083 was highly similar (99.78%) to that of the *Roseobacter* CHAB-I-5 strain *Rhodobacteraceae bacterium* sp. SB2. Phylogenetic analysis of the 16S rRNA gene sequence also showed that strain FZCC0083 was located in the CHAB-I-5 lineage along with SB2 (Supplementary Figure 1). These results indicate that strain FZCC0083 belongs to the CHAB-I-5 lineage.

Two CHAB-I-5 phages, CRP-901 and CRP-902, were isolated from the coastal waters of the North Sea (lat.’ N53°56′, long. E7°48′) and the Yellow Sea (lat.’ N36°38′, long. E121°10′), respectively (Table 1). The genomes of both phages were assembled into a circular contig with a terminal repeat, suggesting the completeness of their genomes. The genome sizes of CRP-901 and CRP-902 are 53.01 kb and 51.95 kb, respectively. The G + C content of CRP-901 and CRP-902 is 45.56 and 45.55%, respectively, similar to that of their host FZCC0083 (48.09%). Despite the large geographic distance between the isolation locations of the two phages, they have similar genome sizes and share 89.17% ANI and 84.73% AAI. Based on ANI classification at the genus level (>70%) and species levels (>95%; Turner et al., 2021), these two phages are different species in the same genus.

### 3.2. Genome characteristics of CHAB-I-5 phages

**Figure 1:**
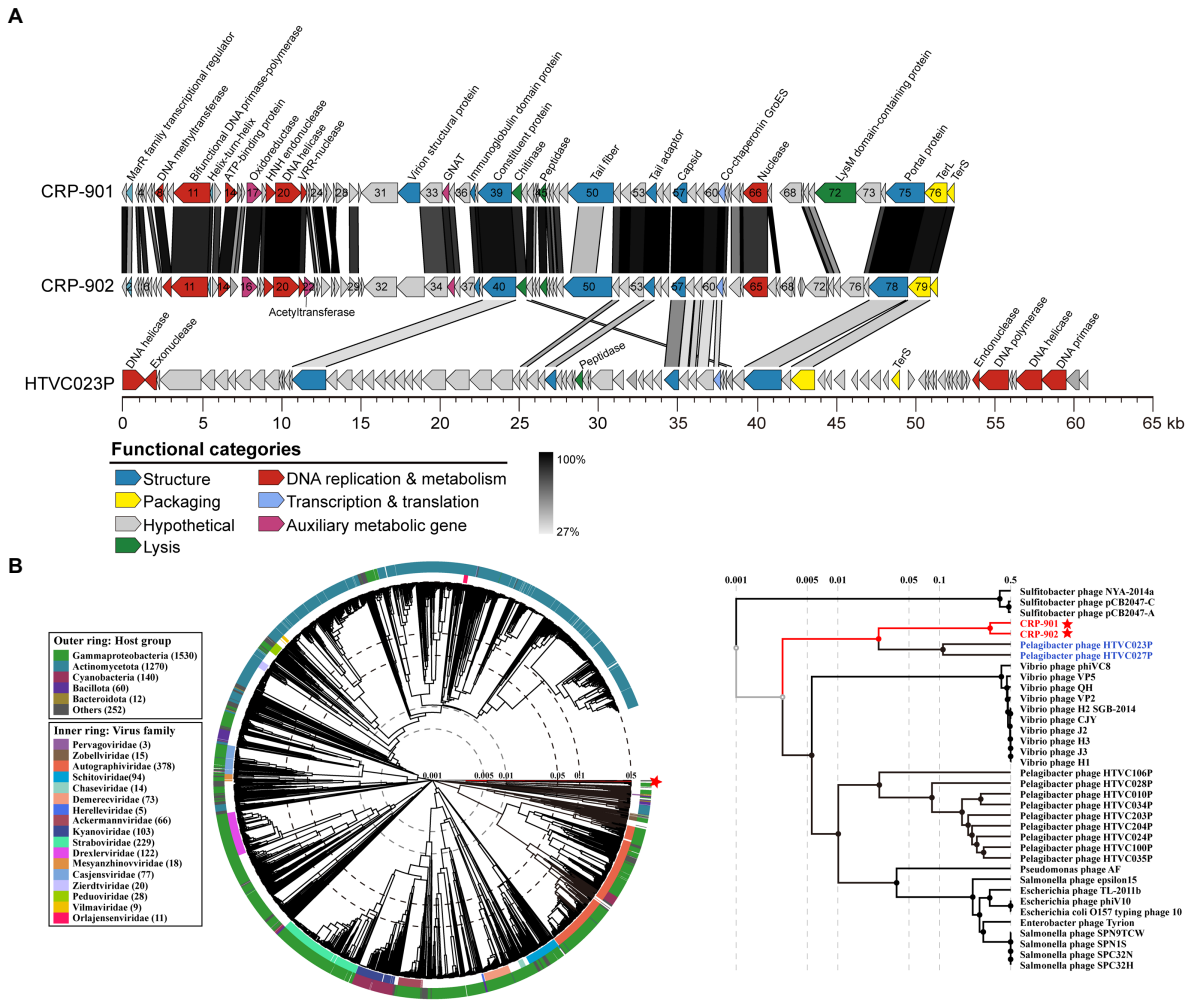
Genomic characterization and phylogenetic analysis of CRP-901 and CRP-902. **(A)** Genome arrangement and comparison of CRP-901, CRP-902 and pelagiphage HTVC023P. ORFs are indicated as arrows and color-coded according to their putative functions. The scale color bar indicates amino acid identities between homologous genes. **(B)** The genome-wide proteomic tree was constructed using VipTree for CRP-901, CRP-902, and other related known prokaryotic dsDNA phages. The colored inner and outer rings represent the virus family and host groups, respectively. CRP-901 and CRP-902 are indicated with red asterisks, and the corresponding leaves are colored red. The pelagiphage HTVC023P and HTVC027P are in blue. TerS, terminase, small subunit; TerL, terminase, large subunit; GNAT, GCN5-Related N-acetyltransferases.

CRP-901 and CRP-902 encode 77 and 80 putative ORFs, respectively. The genomic contents of these two phages are highly similar, and share a total of 59 homologous genes. Most ORFs in both phage genomes showed homology to bacterial or environmental virus sequences. According to the sequence similarity or conserved domains, the putative biological functions of 35 and 31 putative ORFs were annotated in CRP-901 and CRP-902, respectively (Figure 1A; Supplementary Table 4). These annotated genes are mainly related to phage DNA metabolism and replication, phage structure, DNA packaging, and cell lysis. No lysogen-related genes were found in these two phages, suggesting that both phages have a strict lytic life cycle.

The CRP-901 and CRP-902 genomes were roughly divided into two functional modules: a DNA replication/metabolism module and a phage structure and DNA packaging module (Figure 1A). Neither phage has RNA polymerase genes, suggesting that both phages rely on host transcriptional mechanisms. In the DNA replication/metabolism module, both phages contain genes encoding DNA helicase (CRP901_gp20 and CRP902_gp20) and nuclease (CRP901_gp21 and CRP902_gp21), but they do not have DNA primase and DNA polymerase genes, which are required for DNA replication. However, we found a novel bifunctional DNA primase-polymerase gene (CRP901_gp11 and CRP902_gp11) with a bifunctional Prim-Pol domain (PF09250) in both genomes. The bifunctional Prim-Pol domain was shown to possess both primase and polymerase activities (Lipps et al., 2004). The bifunctional DNA primase-polymerase gene has been identified in some phages (Halgasova et al., 2012; Zhu et al., 2017). In phage NrS-1, the bifunctional DNA primase-polymerase gene has been shown to synthesize long-strand DNA directly from DNA templates without a primer binding using dNTPs (Zhu et al., 2017). The bifunctional DNA primase-polymerase genes of CRP-901 and CRP-902 are most similar to that of *Loktanella* phage pCB2051-A, with 38.72% and 40.58% amino acid identity, respectively. The bifunctional DNA primase-polymerase phylogenetic tree showed that these two phages have a distant relationship with *Loktanella* phage pCB2051-A (Supplementary Figure 2A). Although the bifunctional DNA primase-polymerase of these two phages is closest to that of pCB2051-A, other genes in their genomes do not share any homology with those of pCB2051-A. The DNA helicase of CRP-901 and CRP-902 is homologous to that of *Salicola* phage CGphi29, sharing 43.09% and 42.76% amino acid identity, respectively. However, the bifunctional DNA primase-polymerase of the two phages does not share homology with CGphi29 and the two phages share only five genes with CGphi29. The phylogenetic tree of the DNA helicase sequences showed that CRP-901 and CRP-902 form a separate branch from other known viruses (Supplementary Figure 2B). These results indicate that the DNA replication/metabolism modules in CRP-901 and CRP-902 are unique among known viruses.

In the structure and DNA packaging modules of CRP-901 and CRP-902, we identified several genes associated with the tail structure, including genes encoding tail fiber (CRP901_gp50 and CRP902_gp50), tail adaptor (CRP901_gp54 and CRP902_gp54), and portal protein (CRP901_gp75 and CRP902_gp78). The presence of these tail-related genes indicates that the two phages belong to the *Caudoviricetes* class. We noticed that many structural and packaging genes of the two phages shared homology with those of the pelagiphage HTVC023P and HTVC027P (29.63% to 56.52% amino acid identity), including genes encoding capsid protein (CRP901_gp57 and CRP902_gp57), TerL (CRP901_gp76 and CRP902_gp79), and terminase small subunit (TerS, CRP901_gp77 and CRP902_gp80; Figure 1A). HTVC023P and HTVC027 belong to the HTVC023P-type phage group, which is one of the most abundant phage groups in the ocean (Zhang et al., 2021). In the phylogenetic tree of the capsid gene, CRP-901 and CRP-902 are adjacent to the branch of HTVC023P and HTVC027 (Supplementary Figure 2C). By contrast, in the TerL tree, the two phages are adjacent to *Caudovirales* sp. ctOwN3 but occupied a distinct branch from HTVC023P and HTVC027P (Supplementary Figure 2D). Although the structural modules of CRP-901 and CRP-902 are closely related to those in HTVC023P and HTVC027P, their DNA replication genes do not share homology with those in HTVC023P and HTVC027P, indicating that the DNA replicating genes of CRP-901 and CRP-902 have different evolutionary origins and histories from their structural genes.

To determine the phylogenetic relationships between CRP-901, CRP-902, and other related known prokaryotic dsDNA phages, a genome-wide evolutionary tree based on the amino acid sequence was built using the ViPTree server. The tree showed that the two phages form a separate branch adjacent to pelagiphage HTVC023P and HTVC027P (Figure 1B), suggesting that CRP-901 and CRP-902 represent a novel phage group with evolutionary relatedness to pelagiphage HTVC023P and HTVC027P.

### 3.3. MVGs closely related to CRP-901 and CRP-902

**Figure 2:**
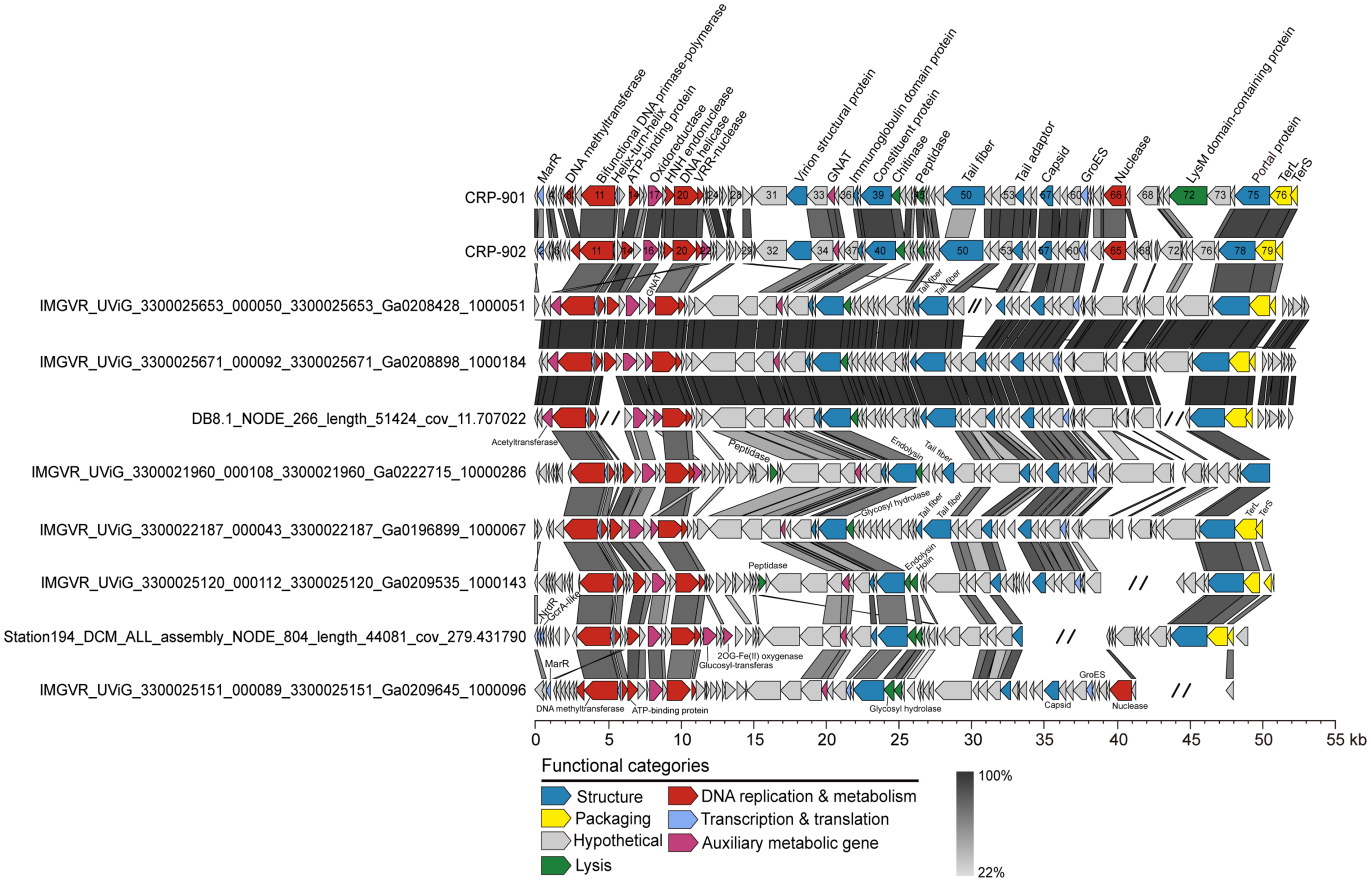
Genome alignment and comparison for CRP-901, CRP-902, and representative CRP-901-type MVGs. ORFs are color-coded according to their putative functions. The scale color bar indicates amino acid identities between homologous genes. MarR, MarR family transcriptional regulator; TerS, terminase, small subunit; TerL, terminase, large subunit; GNAT, GCN5-Related N-acetyltransferases; GroES, Co-chaperonin GroES.

**Figure 3:**
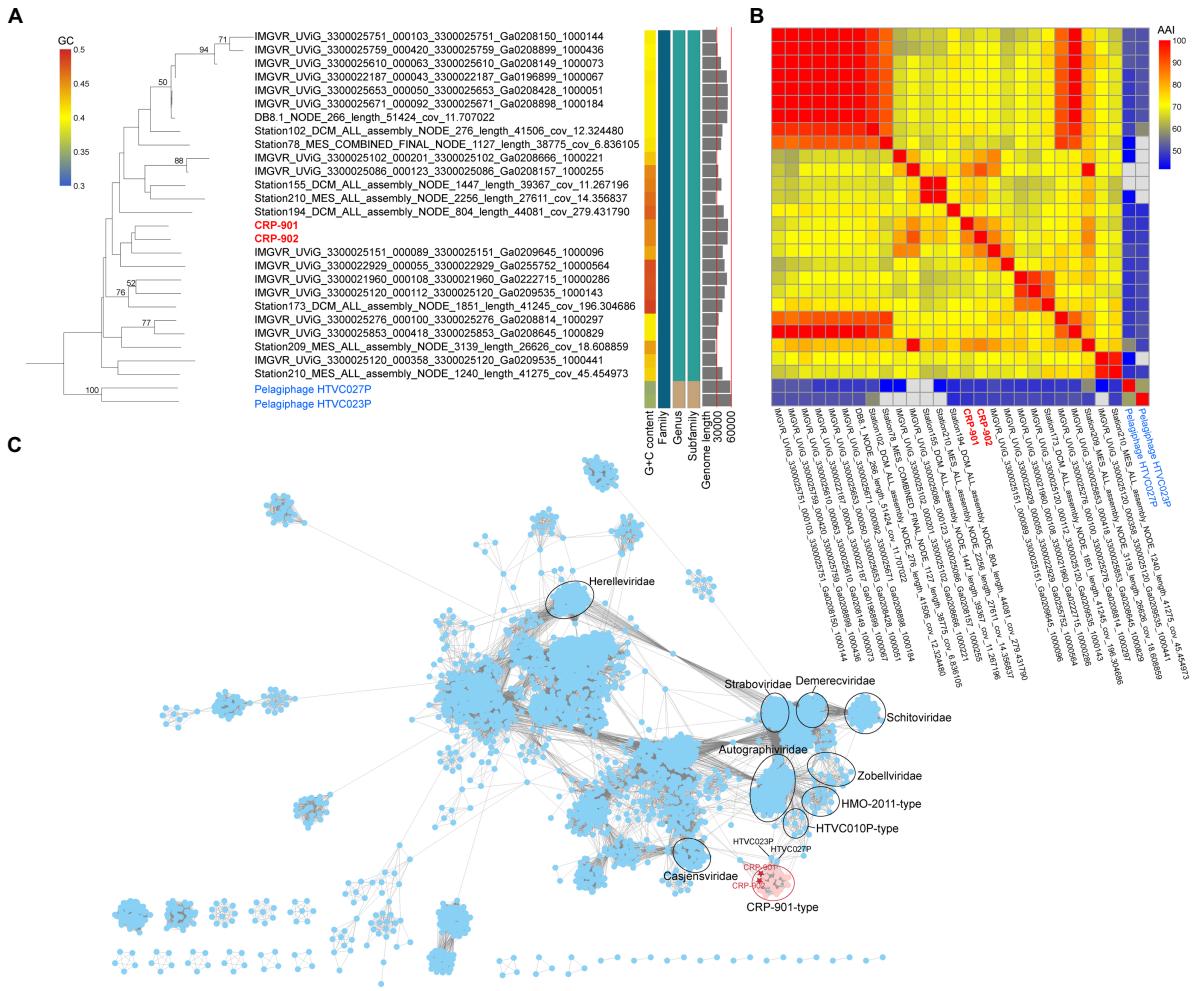
Phylogenetic relationship and viral network of protein content similarity analysis. **(A)** Whole-genome phylogenetic tree based on amino acid sequences constructed by VICTOR with the formula *d_0_* (Meier-Kolthoff and Goker, 2017). CRP-901 and CRP-902 are shown in red, and the pelagiphage HTVC023P and HTVC027P are in blue. The predicted OPTSIL taxon at family, subfamily, and genus are shown as well as the G + C content and sequence length. The bootstrap value of ≥50 is shown on nodes. **(B)** Heatmap showing the average amino acid identity (AAI) value between phage genomes. **(C)** Gene-content-based viral network of CRP-901-type phages and other known viruses from NCBI-RefSeq (v212) constructed by vConTact 2.0. The nodes represent the viruses, and the edges represent the similarity score between two phages, with a cutoff of ≥1. Related phages are circled. CRP-901 and CRP-902 are indicated with red asterisks, and the viral cluster represented by CRP-901 and CRP-902 is shown as a red circle.

To expand our understanding of the biodiversity of the CRP-901-type phages, we performed a metagenomic mining to obtain closely related MVGs from publicly available marine viral metagenomes. A total of 24 MVGs were recovered and used for further analysis. These MVGs are from different aquatic environments, including estuarine, coastal, and open ocean waters (Supplementary Table 5). Genomic analysis revealed that these recovered MVGs exhibit a conserved genome synteny with CRP-901 and CRP-902, with homologous genes located at the same position across the genome (Figure 2). The genome size of these MVGs ranges from 26.63 to 52.46 kb, with 50.79% to 99.47% completeness (Supplementary Table 5). The G + C content of these MVGs ranges from 40.59% to 48.63%, similar to the G + C content of CRP-901 and CRP-902. The AAI values between CRP-901-type phages are greater than 61.34% (Figure 3B).

Most MVGs have DNA helicase, nuclease, and helix-turn-helix transcriptional regulators homologous to CRP-901 and CRP-902 (Figure 2; Supplementary Table 6). Similarly, the DNA primase and DNA polymerase gene were not identified in all MVGs, but the bifunctional DNA primase-polymerase gene homologous to CRP-901 and CRP-902 was identified in most MVGs. These results suggest that these MVGs share a similar DNA replication mechanism with CRP-901 and CRP-902. However, four MVGs were found to encode the RNAP gene, suggesting that CRP-901-type phages may have different transcriptional regulatory mechanisms. The genes encoding capsid, TerS, TerL, tail adaptor, portal, and other structural proteins were also identified in most MVGs, and all of them were homologous to those in CRP-901 and CRP-902. We predicted host information for all MVGs using RaFAH software based on protein content with a machine-learning approach. Of the 26 MVGs, 14 were predicted to be infect CHAB-I-5, 5 were predicted to infect *Planktomarina*, and one was predicted to infect *Pseudomonas*. The potential hosts of the remaining 6 MVGs were unknown (Supplementary Table 5). The host of these MVGs needs further experimental confirmation.

Auxiliary metabolic genes (AMGs) are a class of metabolic genes encoded by phages with similar functions to host-related genes (Breitbart et al., 2007; Suttle, 2007; Breitbart, 2012). AMGs regulate the host’s metabolism and improve its metabolic activity during infection to help phages complete progeny. CRP-901 and CRP-902 contain one and two acetyltransferases, respectively (Figure 1A; Supplementary Table 4). In addition to acetyltransferase, CPR-901 and CRP-902 also have an oxidoreductase gene that is involved in catalyzing biological redox reactions. Similarly, the acetyltransferase gene and oxidoreductase gene were found in the genomes of the 24 MVGs (Supplementary Table 6). The molecular function of acetyltransferase is to catalyze the transfer of acyl groups from acyl-CoA to the amino groups of a wide range of substrates (Salah Ud-Din et al., 2016). Acetyltransferases are involved in various cellular processes, including carbohydrate and energy metabolism, nucleotide and amino acid metabolism, transcription, translation, cell differentiation, and stress regulation. This enzyme is an indispensable part of bacterial metabolism. This result suggests that CRP-901-type phages may regulate the cellular metabolism of the host through phage-encoded acetyltransferase to complete progeny reproduction.

### 3.4. CRP-901-type phages represent a novel genus of bacteriophages

Genome synteny and the high AAI values (61.34%) between CRP-901-type phages indicate that they all belong to the same genus. To further determine the relationship between CRP-901-type phages and evaluate their taxonomic position, a genome-wide phylogenetic tree was constructed using VICTOR (Meier-Kolthoff and Goker, 2017). The genome-wide phylogenetic tree and OPTSIL taxon prediction indicated that all CRP-901-type phages belong to the same genus. They were classified into the same family with pelagiphages HTVC023P and HTVC027P (Figure 3A).

A gene-content-based network and clustering was constructed using vConTACT 2. The CRP-901-type phages were grouped into a viral cluster (VC) distant from other known viruses (Figure 3C). This result is consistent with comparative genomic analysis and VICTOR phylogeny, and further supports that these CRP-901-type phages represent a unique genus-level group to the currently known viruses.

### 3.5. Biogeography of CRP-901-type CHAB-I-5 phages

**Figure 4:**
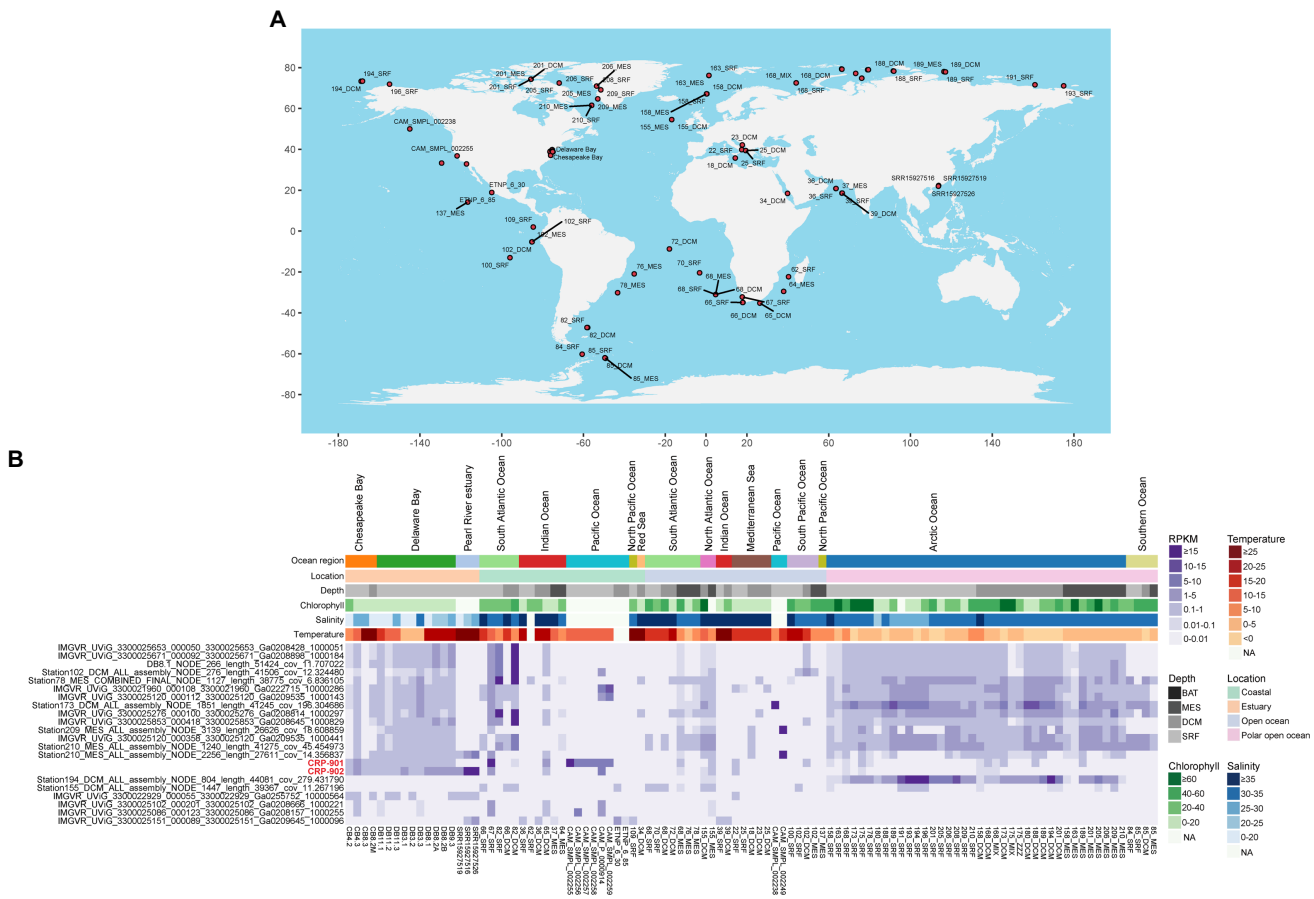
Global distribution and relative abundance of CRP-901-type phages in marine viromes. **(A)** Global distribution profile of the CRP-901-type phage group in marine viromes. The stations where CRP-901-type phages have been detected are shown with red circles. Phages were considered present only if their genome coverage was >20% in the data set. **(B)** The relative abundance of CRP-901-type phages at the species level in different marine viromes. The relative abundance of phages was normalized as the mapped read counts per kilobase pair of genomes per million read counts in the metagenome (RPKM).

We performed viromic read-mapping to analyze the distribution and relative abundance of members in the CRP-901-type phage group. A total of 258 marine virome datasets were used for read-mapping analysis, covering 115 different stations in global oceans (Supplementary Table 3). Among the 258 viromes, CRP-901-type phages were found in 103 viromes, covering oceanic areas from tropical to polar regions (Figure 4A), suggesting that CRP-901-type phages are widely distributed in the ocean.

Although CRP-901 and CRP-902 were isolated from coastal surface water, they were also detected in estuarine and open ocean waters. Both phages were prevalent in estuarine and coastal waters, similar to the distribution of their host CHAB-I-5 (Zhang et al., 2016; Figure 4B). CRP-902 showed the highest RPKM in estuary (*p*-value < 0.05, Mann–Whitney *U*-tests), whereas CRP-901 did not show a significant difference in RPKM between the estuarine and coastal water (*p*-value =0.50, Mann–Whitney *U*-tests). Similar to CRP-901 and CRP-902, 18 of the 20 MVGs were detected prevalent in estuarine waters. Interestingly, 16 MVGs were also prevalent in polar open ocean waters. The RPKM of the 16 MVGs was significantly higher in estuarine and polar open ocean waters than in coastal and non-polar open ocean waters (*p*-value < 0.05, Mann–Whitney *U*-tests), and these MVGs showed the highest RPKM in polar open ocean waters (*p*-value < 0.05, Mann–Whitney *U*-tests). The MVGs that are more abundant in the polar open ocean are mainly from temperate estuarine, coastal waters, and polar open ocean waters (Supplementary Table 5). These results suggest that these MVGs have wide adaptation to temperature and salinity. Linear regression analysis also showed that they are not significantly correlated with temperature and salinity. Of the CRP-901-type phages, only four MVGs were not detected in the polar open ocean waters. The four MVGs showed a very limited distribution and their relative abundance was significantly lower than that of other CRP-901-type phages.

Among different water layers, CRP-901 and CRP-902 were detected in surface, deep chlorophyll maximum, and mesopelagic waters and have higher relative abundance in surface water (*p*-value < 0.05, Mann–Whitney *U*-tests). Similar to CRP-901 and CRP-902, CRP-901-type MVGs were also detected in all three water layers. However, the RPKM values of most MVGs showed no significant difference among different water layers. The distributions of CRP-901 and CRP-902 differed from most CRP-901-type phages, mainly because of their different host adaptations.

**Figure 5:**
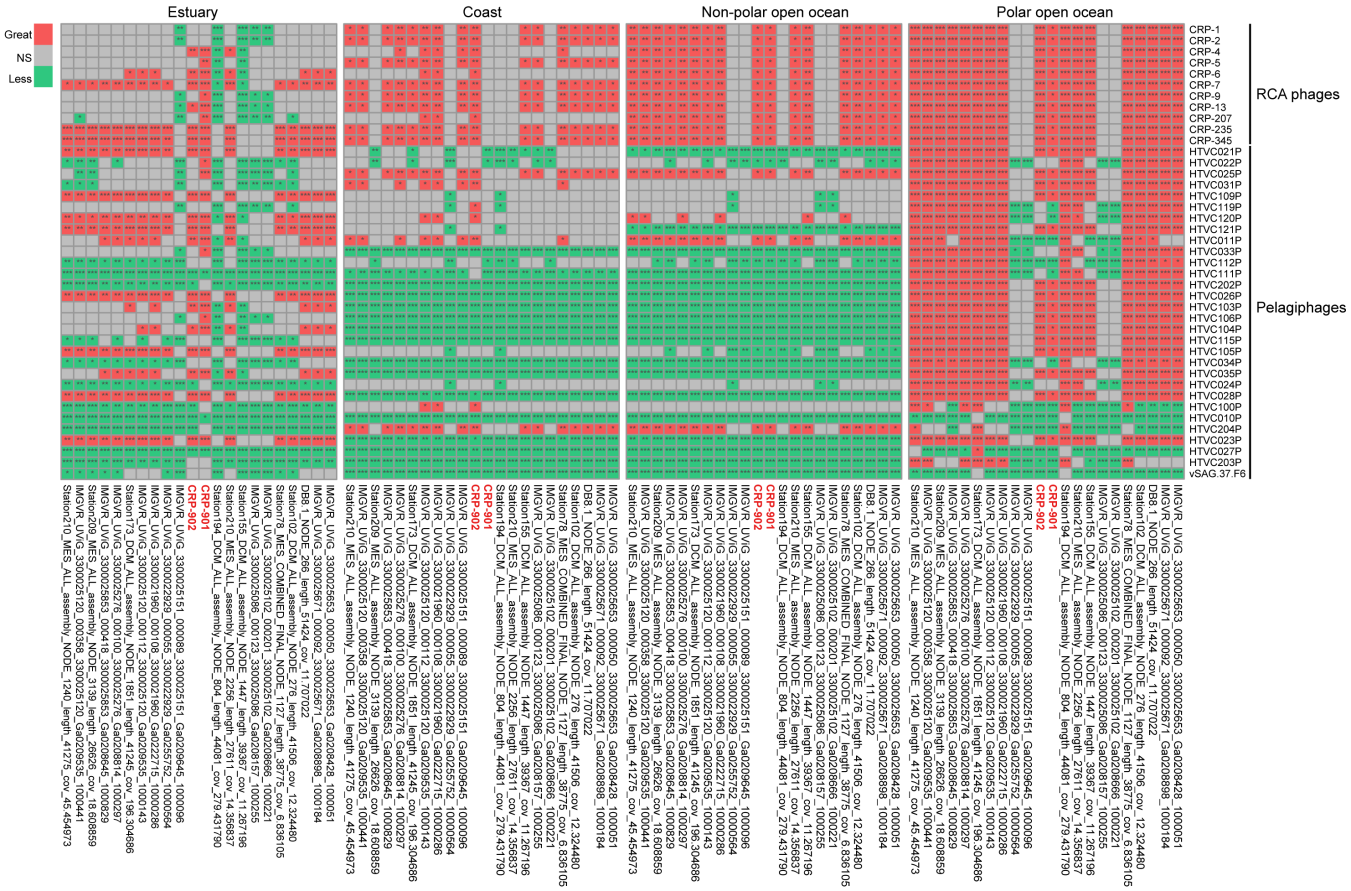
Comparative heatmap of the relative abundance of phages CRP-901-type phages with that of known SAR11 and RCA phages in different marine environments calculated with the two-tailed Mann–Whitney U test. Red, green, and gray indicate significantly greater, significantly less, and not significant, respectively. The significance of pairwise comparisons is indicated using an asterisk corresponding to the *p*-value (*p-value <0.05, **p-value <0.01, ***p-value <0.001). CRP-901 and CRP-902 are labeled in red.

Pelagiphages infecting SAR11 bacteria are generally considered ubiquitous and dominant in the ocean (Zhao et al., 2013; Zhang et al., 2021; Qin et al., 2022). Similarly, RCA phages have also been shown to be universal and abundant in the ocean (Zhang et al., 2019). We compared the relative abundance of CRP-901-type phages with known SAR11 and RCA phages in the ocean. In estuarine waters, most CRP-901-type phages showed a comparable or higher relative abundance with most RCA phages. In coastal and open ocean waters, most CRP-901-type phages were significantly more abundant than all known RCA phages (*p*-value < 0.05; Figure 5). Although the relative abundance of CRP-901-type phages was generally lower than that of most known pelagiphages in the coastal and non-polar open ocean waters, most CRP-901-type phages were significantly more abundant than many known pelagiphages in polar open ocean waters (*p*-value <0.05; Figure 5). These results suggest that CRP-901-type phages are abundant in the ocean and dominate the polar open ocean and estuarine waters.

## 4. Conclusion

This study is the first report of phages infecting the *Roseobacter* CHAB-I-5 strain. Two CHAB-I-5 phages with unique genomic content have been isolated and sequenced. In addition, 24 MVGs closely related to CRP-901 and CRP-902 were recruited from publicly available marine viral metagenomes. Comparative genomic and phylogenetic analyses showed that CRP-901-type phages represent a novel genus in the *Caudoviricetes* class. Further metagenomic analysis showed that they are a widely distributed and abundant phage group in the ocean. This study has expanded the understanding of the genomic diversity, evolution, and ecology of CHAB-I-5 phages and strengthened their ecological importance. The newly isolated CHAB-I-5 phages and their genome sequences will also enable us to further explore their infectivity and ecological strategies, and provide a valuable experimental model for studying the interaction between CHAB-I-5 phages and the host CHAB-I-5.

## Data availability statement

The datasets presented in this study can be found in online repositories. The 16S rRNA gene sequence of strain FZCC0083 have been deposited in the GenBank database under the accession numbers OQ372994. The raw sequencing data of two CHAB-I-5 phages have been deposited in the NCBI Sequence Read Archive under the BioProject accession number PRJNA940680. The genome sequences of CRP-901 and CRP-902 have been deposited in the GenBank database under the accession numbers OQ401623 and OQ401624.

## Author contributions

ZZ: conceptualization, funding acquisition, project administration, methodology, investigation, formal analysis, visualization, data curation, validation, writing—original draft, and writing—review and editing. ZW: methodology, investigation, formal analysis, visualization, and validation. HL and MY: investigation, formal analysis, visualization, and validation. RW: validation and writing—review and editing. YZ and FC: supervision, conceptualization, funding acquisition, project administration, validation, and writing—review and editing. All authors contributed to the article and approved the submitted version.

## Funding

This research was funded by National Key Research and Development Program of China (2018YFA0605800) and National Natural Science Foundation of China (grant number 42076105 and 42206096).

## Conflict of interest

The authors declare that the research was conducted in the absence of any commercial or financial relationships that could be construed as a potential conflict of interest.

## Publisher’s note

All claims expressed in this article are solely those of the authors and do not necessarily represent those of their affiliated organizations, or those of the publisher, the editors and the reviewers. Any product that may be evaluated in this article, or claim that may be made by its manufacturer, is not guaranteed or endorsed by the publisher.

## Supplementary material

SUPPLEMENTARY FIGURE S1 16S rRNA gene phylogenetic tree of FZCC0083 and other known roseobacters. FZCC0083 are labeled in red, and the outgroups are indicated in blue. Related *Roseobacter* lineages are colored.

SUPPLEMENTARY FIGURE S2 Maximum likelihood phylogenetic trees of DNA helicase **(A)**, Bifunctional DNA primase-polymerase **(B)**, capsid **(C)**, and TerL **(D)**.

SUPPLEMENTARY DATA SHEET 1 Hidden Markov model (HMM) profile of four conserved genes of CRP-901 and CRP-902.
