# Supplementary figures and images for: Genomic analysis and characterization of phages infecting the marine Roseobacter CHAB-I-5 lineage reveal a globally distributed and abundant phage genus
Source: Front Microbiol. 2023 Apr 17;14:1164101. doi: 10.3389/fmicb.2023.1164101 (PMC10149686; doi:10.3389/fmicb.2023.1164101)

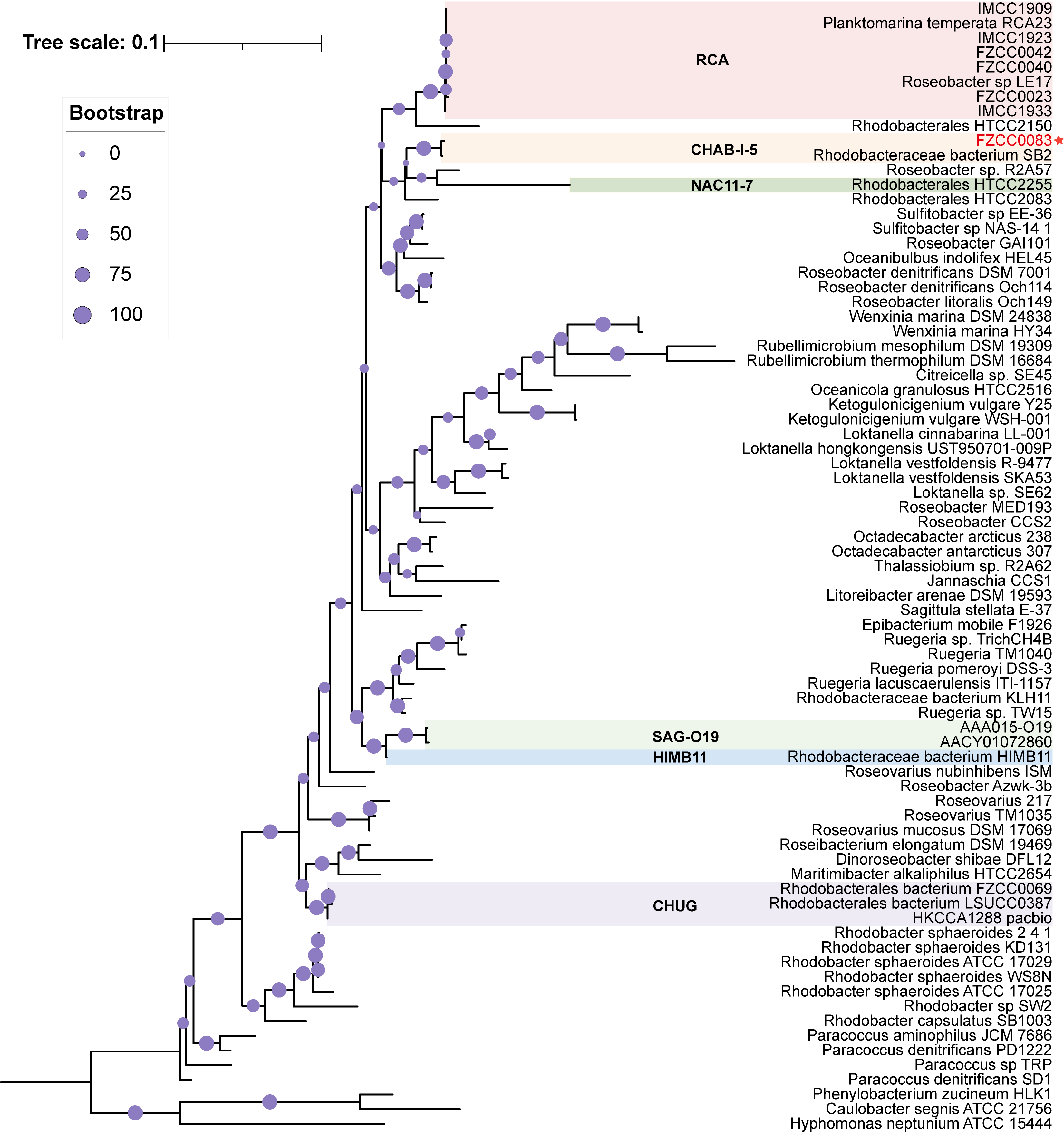

Supplement: SUPPLEMENTARY FIGURE S1 — 16S rRNA gene phylogenetic tree of FZCC0083 and other known roseobacters. FZCC0083 are labeled in red, and the outgroups are indicated in blue. Related Roseobacter lineages are colored. [file Image_1.TIF]

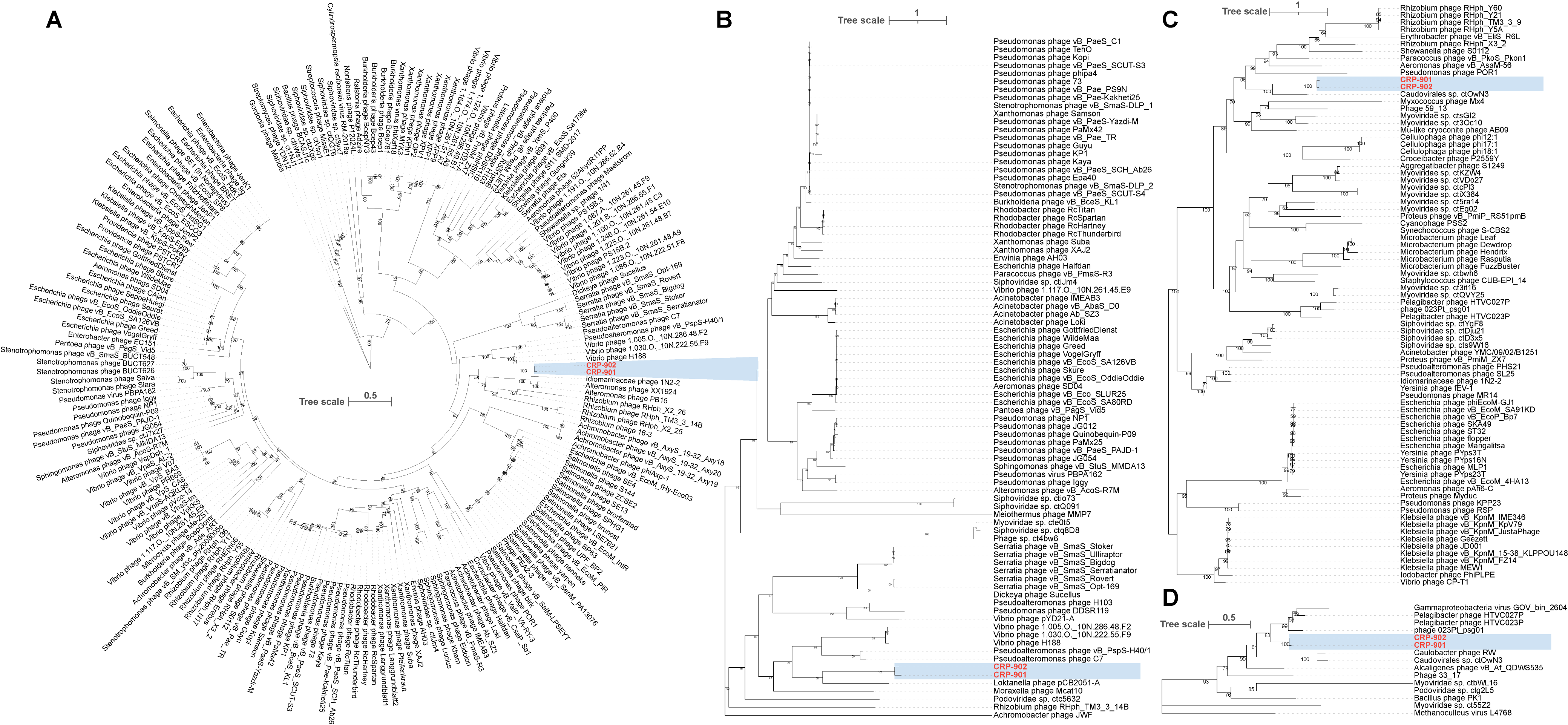

Supplement: SUPPLEMENTARY FIGURE S2 — Maximum likelihood phylogenetic trees of DNA helicase (A), Bifunctional DNA primase-polymerase (B), capsid (C), and TerL (D). [file Image_2.TIF]
